# Impact of liver fibrosis and nodules formation on hemodynamics in young adults after total cavopulmonary connection. A magnetic resonance study

**DOI:** 10.3389/fcvm.2022.986653

**Published:** 2022-09-29

**Authors:** Václav Chaloupecký, Denisa Jičínská, Viktor Tomek, Ondřej Materna, Roman Gebauer, Rudolf Poruban, Petra Antonová, Theodor Adla, Matěj Štefánek, Vojtěch Illinger, Karel Kotaška, Jan Janoušek

**Affiliations:** ^1^Children’s Heart Centre, Second Faculty of Medicine, Charles University in Prague and Motol University Hospital, Prague, Czechia; ^2^Department of Cardiovascular Surgery, Second Faculty of Medicine, Charles University in Prague and Motol University Hospital, Prague, Czechia; ^3^Department of Radiology, Second Faculty of Medicine, Charles University in Prague and Motol University Hospital, Prague, Czechia; ^4^Department of Rehabilitation and Sports Medicine, Second Faculty of Medicine, Charles University in Prague and Motol University Hospital, Prague, Czechia; ^5^Department of Medical Chemistry and Clinical Biochemistry, Second Faculty of Medicine, Charles University in Prague and Motol University Hospital, Prague, Czechia

**Keywords:** Fontan procedure, liver fibrosis, liver nodules, ELF test, procollagen III amino-terminal peptide, magnetic resonance imaging

## Abstract

**Background:**

The aim of this study was to analyze the relation between the hepatic fibrosis markers, liver morphology and hemodynamics assessed by magnetic resonance imaging (MRI) after total cavopulmonary connection (TCPC).

**Materials and methods:**

Adult patients after TCPC performed in childhood between 1993 and 2003 are the subjects of this observational study. The follow-up protocol consisted of clinical and echocardiographic examination, liver elastography, cardiopulmonary exercise test, MRI hemodynamics and liver morphology assessment and direct enhanced liver fibrosis (ELF) test.

**Results:**

The cohort consisted of 39 patients (46% female) with a median age at study 26 (IQR 23–28) years and interval from TCPC 21 (IQR 20–23) years. There was no correlation between ELF test and any MRI variables, but procollagen III amino-terminal peptide (PIIINP), a single component of ELF test, correlated significantly with ventricular end-diastolic volume (*r* = 0.33; *p* = 0.042) and inferior vena cava flow (*r* = 0.47; *p* = 0.003). Fifteen (38%) patients with liver nodules had compared to other 24 patients higher end-diastolic volume (ml/m^2^) 102.8 ± 20.0 vs. 88.2 ± 17.7; *p* = 0.023, respectively. PIIINP correlated significantly with inferior vena cava flow (*r* = 0.56; *p* = 0.030) and with end-diastolic volume (*r* = 0.53; *p* = 0.043), but only in patients with liver nodules.

**Conclusion:**

Gradual progression of liver fibrosis, particularly hepatic arterialization caused by liver nodules formation, increases inferior vena cava flow and subsequent ventricular volume overload may further compromise single ventricle functional reserve in adult patients after TCPC.

## Introduction

For the last decades, the total cavopulmonary connection (TCPC) provides the best palliative procedure improving prognosis of many children born with functionally univentricular heart disease (1, 2). By diverting systemic venous blood to the pulmonary arteries TCPC eliminates mixing of oxygenated and deoxygenated blood and ventricular volume overload caused by intracardiac shunts and establishes almost normal arterial blood oxygenation but with resulting elevated systemic venous pressure. The 20-year survival rate in a recent multicenter epidemiological study 87.9 and 93.0% for lateral and extracardiac type of TCPC, respectively is encouraging (3), especially when compared to the grim natural survival of these complex heart anomalies. The long-term prognosis of patients with univentricular circulation seems to be, however, almost universally jeopardized by the Fontan associated liver disease (FALD). FALD is a form of congestive hepatopathy related to the increased systemic venous pressure and borderline cardiac output caused by energy loss within the TCPC system and intrinsic malfunctions of the univentricular heart (4–6). The aim of this single institutional study was to analyze the relation between univentricular Fontan hemodynamics assessed by magnetic resonance imaging (MRI) and the direct biochemical markers of liver fibrosis and liver morphology in adult patients after TCPC performed in their childhood.

## Materials and methods

### Study population

Out of 215 children who underwent TCPC at Children’s Heart Centre for various univentricular congenital heart defects between 1993 and 2003, a total of 39 adult patients are the subjects of this observational study performed between years 2016 and 2018 at Motol University Hospital. The institutional TCPC follow-up protocol consisted of clinical and echocardiographic examination, liver ultrasound and elastography, cardiopulmonary exercise test (CPET), MRI evaluation of the heart function, blood flow measurements and liver morphology and routine biochemical liver tests. A quality of life was assessed by SF-36 questionnaire. The perioperative risk factors were reviewed from historical clinical, catheterization and echocardiographic records and overall TCPC risk score was calculated according to Fisher et al. (7). The study was approved by the Motol University Hospital Ethics Committee (EK-1556/16). An informed consent was not required, as all data were collected as part of the institutional follow-up protocol.

### Cardiopulmonary exercise test

All CPETs were performed on an electromagnetically braked bicycle ergometer (Ergoline Ergoselect 100, Ergoline GmbH, Germany). The maximal incremental stepwise protocol was used in all tests until patient exhaustion. Breath by breath measurement of gas exchange and ventilation was monitored by a calibrated gas exchange analyzer (Oxycon Pro with the electrochemical oxygen sensor, Jaeger, Germany). Each test result was checked for consistency in the physiological response of the data by an exercise physiologist (8).

### Cardiac magnetic resonance imaging acquisition

All examinations were performed in a fasting state on 1.5T MRI machine (Avanto, Siemens, Germany). The protocol included static steady-state free procession (SSFP) in axial, coronal and sagittal planes, cine SSFP in long axes and short axis. 3D SSFP, and contrast-enhanced time-resolved 3D MR angiography, pre and post contrast T1 VIBE (volumetric interpolated breath-hold examination) Dixon sequences. The volumes and ejection fractions were calculated from consecutive cine SSFP retrospectively gated images on a short axis (slice thickness 8 mm, gap 2 mm, 25 phases). Manual delineation of the endocardial borders was performed in end-diastole and end-systole.

Phase-contrast velocity encoded images for flow quantification were obtained in multiple localizations: above aortic or neoaortic valve, when two outlets were present, they were measured separately and summed as aortic flow (Qao), inferior vena cava flow (Qivc), superior vena cava flow (Qsvc), right (Qrpa) and left (Qlpa) pulmonary artery flow and right (Qrpv), and left (Qlpv) pulmonary venous flow. TCPC flows indexed to body surface area (BSA) were calculated according to the method published by Whitehead as follows (9). Pulmonary venous blood flow (Qp) = (Qrpv) + (Qlpv), systemic venous blood flow (Qs) = (Qsvc) + (Qivc). Aortopulmonary collateral flow was calculated as the difference between pulmonary venous blood flow and pulmonary arterial blood flow as (Qcoll-pulm) = (Qp)–(Qpa). A dedicated workstation with the Syngo software (Siemens, Germany) was used for volume and flow measurements and values were indexed for the BSA.

Liver morphology was assessed for any abnormalities using pre and post-contrast T1 images, MR angiography which covered the chest and upper abdomen including the liver, and partially also from 3D SSFP images.

### Liver ultrasound examination and elastography

Liver stiffness (in κPa) was measured by shear-wave elastography using Toshiba/Canon Aplio 500. The scanning was performed in the supine position and the subjects were asked to hold their breath to avoid motion and deep inspiration. A single-shot 2D elastographic map was created and the operator picked a representative 1 cm wide circular region of interest (ROI) of the liver tissue at least 1 cm deep in the 7th liver segment to avoid subcapsular measurement. The final value of SW elastography is the median value of 10 measurements. Routine B-mode ultrasound of the liver was performed. The size and shape of the liver, contours, echogenicity, echotexture and presence of liver lesions were assessed.

### Laboratory evaluation

The following laboratory parameters were evaluated: routine liver biochemical tests, total protein, albumin, prealbumin (Advia Chemistry system), N-terminal pro-brain natriuretic peptide (Roche Cobas Platform), hepatitis A, B, and C serology (Abbott Architect platform). The ELF test consisting of hyaluronic acid (HA), procollagen III amino-terminal peptide (PIIINP), and tissue inhibitors of matrix metaloproteinases (TIMP-1) analyses were assessed using the ADVIA Centaur platform. ELF test score was calculated using formula ELF score = 2.278 + 0.851 ln(cHA) + 0.751 ln(cPIIINP) + 0.394 ln(cTIMP-1). The score has a cut-off ⇒ 7.7 for the presence of fibrosis (10).

### Statistical analysis

Normally distributed variables were reported as mean ± standard deviation, skewed variables as median and interquartile range (25th–75th percentile) and nominal data as count and percentage (%). Independent *t*-test, or non-parametric tests were used where applicable to compare continuous variables. Fisher’s exact test was used to compare categorical variables. Correlations were tested by Pearson’s correlation coefficient if both groups were normally distributed, otherwise a Spearman’s correlation was used. A two-sided level of *P*-value < 0.05 was considered statistically significant. Data were analyzed using IBM SPSS software (version 28.0, SPSS Inc, Armonk, USA).

## Results

### Patient characteristic

The cohort consisted of 39 patients with a median age at study 26 (IQR 23–28) years and interval from TCPC 21 (IQR 20–23) years. Patient clinical data are summarized in Table 1. The main diagnoses were double inlet left ventricle in 10 patients, tricuspid atresia in 10 patients, double outlet right ventricle or transposition of the great arteries or with hypoplastic ventricle and/or straddling AV valve in 10 patients, double inlet right ventricle in 3 patients and other in remaining 6 patients. Overall 30 (77%) patients had dominant left ventricular morphology. Twenty (52%) patients underwent previous modified Blalock–Taussig shunt, 7 (18%) pulmonary artery banding, 7 (18%) bidirectional cavopulmonary anastomosis and 2 patients myectomy and 2 patients Damus–Kaye–Stansel procedure for a systemic outflow tract obstruction. Thirty-six (92%) patients underwent TCPC with lateral tunnel. The fenestration created in 12 (31%) patients was subsequently closed at catheterization in 11 cases or closed spontaneously in 1 patient. Atrioventricular valve regurgitation was graded at echocardiographic examination as none to mild in 37 (95%) patients, semilunar valve regurgitation as none to mild in all 39 patients and overall heart function as good or mildly depressed in 37 (95%) patients. All patients were in New York Heart Association (NYHA) functional class I or II. One patient was on warfarin and all others were on antiplatelet therapy. A minor thromboembolic event occurred in 1 patient from a residual pulmonary artery stump.

**TABLE 1:** Patient characteristic (*n* = 39).

	Values
Gender–female	18 (46%)
TCPC type (lateral tunnel/extracardiac)	36/3
Fenestration	12 (31%)
Subsequent fenestration closure	12
Ventricular morphology (left/right or indeterminate)	30 (77%)
**NYHA class**	
I	28 (72%)
II	11 (28%)
**Ventricular function (echocardiography)**	
Good	32 (82%)
Mildly depressed	5 (13%)
Moderately depressed	2 (5%)
**Atrioventricular valve regurgitation**	
None to trivial	27 (69%)
Mild	10 (26%)
Moderate	2 (5%)
**Semilunar valve regurgitation**	
None to trivial	35 (90%)
Mild	4 (10%)

Data are represented as n (%).

TCPC, total cavopulmonary connection.

The results of biochemical tests in 39 patients are summarized in Table 2. Gamma glutamyl transferase (GGT) was abnormally elevated in 82% and bilirubin in 46% of patients. The ELF score ≥ 7.7 indicating the liver fibrosis was present in 36 (92%) patients.

**TABLE 2:** Biochemical tests in 39 patients at the study.

Variable	Value	Abnormal *N* (%)
GGT (μkat/L)	1.28 (0.89–2.13)	>32 (82%)
AST (μkat/L)	0.49 ± 0.14	>3 (8%)
ALT (μkat/L)	0.52 ± 0.19	>5 (13%)
Bilirubin (μmol/L)	16.6 (11.5–21.2)	>18 (46%)
Total protein (g/L)	74.5 ± 6.5	0
Albumin (g/L)	47.5 ± 3.6	0
Prealbumin (g/L)	0.26 ± 0.06	<2 (5%)
NTproBNP (ng/L)	133.5 ± 99.7	>14 (36%)
ELF test score	8.6 ± 0.50	>36 (92%)
PIIINP (μg/L)	9.2 ± 3.4	10 (26%)
HA (μg/L)	20.6 (14.3–28.8)	NA
TIMP-1 (μg/L)	200.9 (179.9–220.7)	NA

Data are represented as mean ± SD or median (IQR) as appropriate and by *N* (%) of patients with values >above or <below normal limits.

ALT, alanine aminotransferase; AST, aspartate aminotransferase; GGT, gamma glutamyl transferase; HA, hyaluronic acid; NA, not applicable; PIIINP, procollagen III amino-terminal peptide; NTproBNP, N-terminal pro-brain natriuretic peptide; TIMP-1, tissue inhibitors of matrix metaloproteinases.

### Enhanced liver fibrosis test and magnetic resonance imaging parameters

There was no correlation between any MRI parameter and the ELF test (Supplementary Table 1). When ELF test components were analyzed separately, only PIIINP correlated significantly with ventricular end-diastolic volume (*r* = 0.33; *p* = 0.042), stroke volume (*r* = 0.34; *p* = 0.034), systemic venous flow (*r* = 0.45; *p* = 0.005), and inferior vena cava flow (*r* = 0.47; *p* = 0.003). Therefore only PIIINP was used for further analyses as an independent variable of the liver fibrosis.

### Procollagen III amino-terminal peptide, clinical data, and magnetic resonance imaging parameters

There was no correlation between clinical and hemodynamic data at TCPC and PIIINP level at the study, except for cardiopulmonary bypass time (Table 3). The type of previous procedures, single ventricular morphology, degree of atrioventricular regurgitation and fenestration at TCPC had no effect on PIIINP at the study.

**TABLE 3:** Correlations between clinical and hemodynamic data at total cavopulmonary connection (TCPC) and procollagen III amino-terminal peptide (PIIINP) at study.

	Value	*r*	*P*-value
Age (yrs)	4 (3–6)	–0.243	0.136
Body weight (kg)	15.9 (12.8–21.0) 8.0	–0.219	0.181
BSA (m^2^)	0.69 (0.59–0.89)	–0.226	0.166
Mean pulmonary artery pressure (mm Hg)	12.0 ± 4.0	0.068	0.681
Pulmonary vascular resistance (WU × m^2^)	1.9 ± 0.9	0.28	0.869
McGoon index	2.3 ± 0.5	–0.201	0.226
Nakata index (mm^2^/m^2^)	374 ± 140	–0.190	0.253
Risk score	4 (2–5)	0.098	0.553
Cardiopulmonary bypass (min)	139 ± 29	0.344	0.032
**Early post-operative period**			
Central venous pressure (mm Hg)	12 ± 2	0.239	0.155
Transpulmonary gradient (mm Hg)	7 ± 2	–0.232	0.195

Data are represented as mean ± SD or median (IQR) and Pearson’s or Spearman’s coefficients as appropriate.

BSA, body surface area; TCPC, total cavopulmonary connection.

The correlation between clinical data and PIIINP blood levels at the study are summarized in Table 4. There was no significant correlation between PIIINP and age at study, age at TCPC or at interval from TCPC, liver elastography, maximal oxygen consumption at exercise, and pulse oximetry at rest or at exercise.

**TABLE 4:** Correlations between clinical data and procollagen III amino-terminal peptide (PIIINP) at study.

	Value	*r*	*P*-value
Age (yrs)	26 (23–28)	–0.253	0.120
Interval from TCPC (yrs)	21 (20–23)	–0.077	0.640
Body weight (kg)	69.1 ± 14.3	0.226	0.167
BMI (kg m^–2^)	22.0 (20.1–26.3)	0.191	0.244
Liver elastography	13.7 (12.4–15.7)	–0.039	0.821
VO_2*max*_ (mL/min/kg)	28.6 ± 5.6	–0.054	0.750
SPO_2_ resting (%)	96 (94–97)	0.029	0.861
SPO_2_ at VO_2*max*_ (%)	87 (85–89)	–0.239	0.142

Data are represented as mean ± SD or median (IQR) and Pearson’s or Spearman’s coefficients as appropriate.

BMI, body mass index; SPO_2_, pulse oximetry; TCPC, total cavopulmonary connection; VO_2*max*_, maximal oxygen consumption during exercise.

**FIGURE 1:**
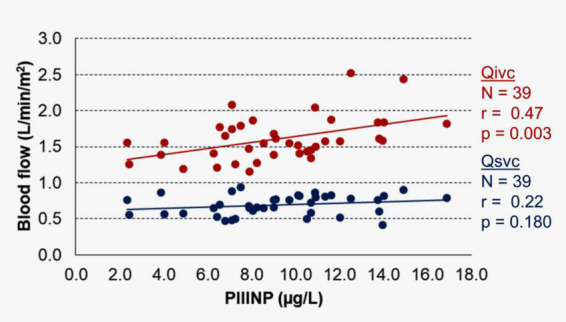
Correlation between procollagen III N-terminal peptide (PIIINP) and inferior vena cava (Qivc) or superior vena cava (Qsvc) flow.

The effect of PIIINP level on the systemic venous blood flow return in all 39 patients measured by MRI is shown in Figure 1. There was a significant correlation between PIIINP and inferior vena cava flow (Qivc), but not between PIIINP and superior vena cava flow (Qsvc).

### Association between liver nodules, clinical data, biochemical tests, and magnetic resonance imaging parameters

Liver nodules were observed on MRI or on sonographic examination in 15 (38%) out of 39 patients. The nodules were round, frequently multiple (in 7 patients more than one), peripherally located and often hyperenhancing in the arterial phase. For further analysis, subjects were divided into two subgroups according to the presence or absence of liver nodules.

There was no significant difference in clinical and catheterization data and perioperative data at TCPC between patients with and without liver nodules, except for elevated central venous pressure in the early post-operative period (*p* = 0.020) (Table 5). There was also no difference between both subgroups in clinical data and biochemical tests at the time of study, except for increased PIIINP (*p* = 0.009) in patients with detected liver nodules (Table 6).

**TABLE 5:** Occurrence of liver nodules at the time of study and clinical data at total cavopulmonary connection (TCPC).

Variable	Present (*N* = 15)	Absent (*N* = 24)	*P*-value
Age (yrs)	3.5 (3.0–4.0)	5.5 (3.2–6.8)	0.161
Body weight (kg)	14.0 (12.1–18.0)	16.9 (13.3–23.9)	0.166
Left ventricular morphology	11 (73%)	19 (79%)	0.711
Mean pulmonary artery pressure (mm Hg)	12 ± 3	12 ± 5	0.940
Pulmonary vascular resistance (WU × m^2^)	1.9 ± 1.0	1.9 ± 0.8	0.521
McGoon index	2.2 ± 0.4	2.3 ± 0.6	0.554
Nakata index (mm^2^/m^2^)	370 ± 111	376 ± 157	0.364
Risk factor	4.0 ± 2.0	3.4 ± 1.9	0.364
Cardiopulmonary bypass time (min)	132 ± 28	143 ± 30	0.224
**Early post-operative period**			
Central venous pressure (mm Hg)	13 ± 1	12 ± 2	0.020
Transpulmonary gradient (mm Hg)	6 ± 1	7 ± 2	0.298
Chest drainage (days)	6 (5–10)	5 (4–8)	0.705

Data are represented as mean ± SD or median (IQR) as appropriate.

TCPC, total cavopulmonary connection.

**TABLE 6:** Liver nodules, clinical data, and laboratory tests at the study.

Variable	Present (*N* = 15)	Absent (*N* = 24)	*P*-value
Age (yrs)	23.5 (22.4–27.5)	26.3 (24.6–28.1)	0.083
Interval from TCPC (yrs)	21.0 (19.1–22.2)	21.6 (20.2–22.6)	0.503
Body weight (kg)	68.2 ± 13.7	69.6 ± 14.9	0.758
BMI (kg m^–2^)	22.0 (20.1–26.3)	21.9 (19.8–26.6)	0.980
Liver elastography (κPa)	13.7 (12.8–14.7)	13.4 (12.1–15.7)	0.688
VO_2*max*_ (mL/min/kg)	29.1 ± 4.6	28.3 ± 6.4	0.661
SPO_2_ resting (%)	96 (94–97)	96 (95–97)	0.517
SPO_2_ at VO_2*max*_ (%)	86 (85–89)	88 (85–89)	0.925
Quality of life (SF-36)	85 (80–89)	83 (77–88)	0.443
GGT (μkat/L)	1.7 (1.1–2.7)	1.2 (0.7–1.6)	0.103
AST (μkat/L)	0.50 ± 0.10	0.48 ± 0.15	0.544
ALT (μkat/L)	0.50 ± 0.16	0.53 ± 0.20	0.665
Bilirubin (μmol/L)	14.0 (11.5–21.2)	17.2 (11.8–21.8)	0.697
Total protein (g/L)	74.2 ± 6.1	74.7 ± 6.9	0.815
Albumin (g/L)	48.3 ± 4.5	47.0 ± 2.9	0.297
Prealbumin (g/L)	0.25 (0.23–0.32)	0.24 (0.22–0.28)	0.385
NTproBNP (ng/L)	90.5 (74.9–166.7)	103.4 (60.6–183.0)	0.633
ELF score	8.8 ± 0.3	8.5 ± 0.6	0.056
PIIINP (μg/L)	11.0 ± 3.4	8.1 ± 2.9	0.009
HA (μg/L)	20.7 (16.2–27.3)	18.6 (13.0–28.8)	0.593
TIMP-1 (μg/L)	195.8 (182.0–220.7)	201.5 (177.6–222.2)	0.762

Data are represented as mean ± SD or median (IQR) as appropriate.

ALT, alanine aminotransferase; AST, aspartate aminotransferase; BMI, body mass index; ELF, enhanced liver fibrosis test; GGT, gamma glutamyl transferase; HA, hyaluronic acid; NTproBNP, N-terminal pro-brain natriuretic peptide; PIIINP, procollagen III amino-terminal peptide; SPO_2_, pulse oximetry; TCPC, total cavopulmonary connection; TIMP-1, tissue inhibitors of matrix metaloproteinases; VO_2*max*_, maximal oxygen consumption at exercise.

Association between the presence of liver nodules and MRI hemodynamic parameters is summarized in Table 7. Patients with liver nodules had significantly higher end-diastolic volume (*p* = 0.023), stroke volume (*p* < 0.001), aortic blood flow (*p* = 0.022), systemic venous flow (*p* = 0.036), and lower heart rate. A significant negative correlation between heart rate and end-diastolic volume was confirmed only in subgroup of patients without nodules (*r* = 0.62; *p* = 0.001), but not in patients with nodules (*r* = 0.42; *p* = 0.123). There was no difference in both groups between an atrioventricular regurgitation fraction and aortopulmonary collateral flow.

**TABLE 7:** Liver nodules and magnetic resonance imaging (MRI) parameters.

Variable	Present (*N* = 15)	Absent (*N* = 24)	*P*-value
Heart rate (beats/min)	66 ± 9	75 ± 14	0.034
End-diastolic volume (mL/m^2^)	102.8 ± 20.0	88.2 ± 17.7	0.023
End-systolic volume (mL/m^2^)	41.6 (36.3–62.3)	38.8 (34.0–49.1)	0.279
Stroke volume (mL/m^2^)	54.1 ± 8.4	45.0 ± 7.1	<0.001
Cardiac index (L/min/m^2^)	3.3 (3.1–3.8)	3.2 (2.9–3.5)	0.225
Ejection fraction	0.54 ± 0.08	0.52 ± 0.08	0.554
AVVRf (%)	10 ± 6	12 ± 9	0.343
End-diastolic myocardial mass (g/m^2^)	49.5 ± 19.1	51.2 ± 13.2	0.750
AO flow (L/min/m^2^)	3.1 ± 0.5	2.8 ± 0.4	0.022
Systemic venous flow (L/min/m^2^)	2.5 ± 0.5	2.2 ± 0.3	0.036
IVC flow (L/min/m^2^)	1.7 ± 0.4	1.5 ± 0.2	0.071
SVC flow (L/min/m^2^)	0.7 ± 0.1	0.7 ± 0.1	0.074
IVC/SVC flow	2.3 (2.0–2.7)	2.4 (2.0–2.6)	0.634
Pulmonary artery flow (L/min/m^2^)	2.4 ± 0.5	2.2 ± 0.4	0.718
Pulmonary venous (L/min/m^2^)	3.0 ± 0.6	2.8 ± 0.4	0.257
Qcoll-pulm (L/min/m^2^)	0.6 ± 0.3	0.5 ± 0.2	0.424
Qcoll-pulm/AO flow × 100 (%)	0.19 ± 0.08	0.19 ± 0.07	0.934
McGoon index	2.4 ± 0.7	2.5 ± 0.7	0.674
Nakata index (mm^2^/m^2^)	236 ± 63	251 ± 51	0.401

Data are represented as mean ± SD or median (IQR) as appropriate.

AO, aorta; AVVRf, atrioventricular valve regurgitation fraction; HA, hyaluronic acid; IVC, inferior vena cava; LPA, left pulmonary artery; PIIINP, procollagen III amino-terminal peptide; Qcoll-pulm, aortopulmonary collateral flow; RPA, right pulmonary artery; RPV, right pulmonary vein; SVC, superior vena cava; TIMP-1, inhibitors of matrix metaloproteinases.

**FIGURE 2:**
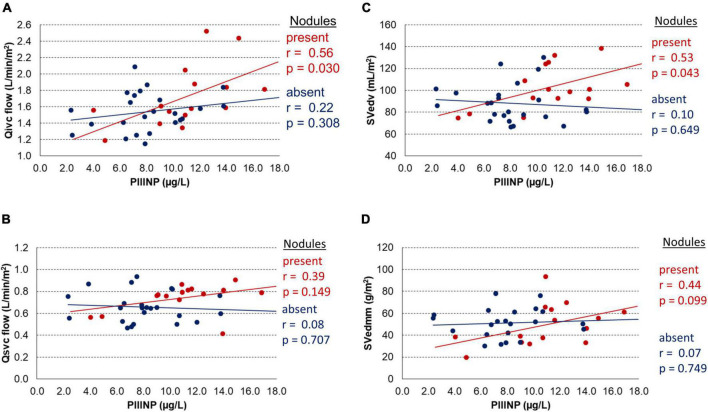
Effects of procollagen III N-terminal peptide (PIIINP) stratified by the presence of liver nodules on: **(A)** inferior vena cava flow (Qivc), **(B)** superior vena cava flow (Qsvc), **(C)** end-diastolic volume (SVedv), **(D)** end-diastolic myocardial mass (SVedmm).

The relation between PIIINP and MRI hemodynamic parameters is shown in Figure 2. There was a significant correlation between PIIINP and inferior vena cava flow (Qivc) but only in the subgroup of patients with liver nodules (*r* = 0.56; *p* = 0.030) (Figure 2A). PIIINP level had no effect on superior vena cava flow (Qsvc) in both subgroups (Figure 2B). The end-diastolic volume increased proportionally to PIIINP blood level, but only in a subgroup of patients with liver nodules (*r* = 0.53; *p* = 0.043) (Figure 2C). PIIINP had no significant effect on end-diastolic myocardial mass in both groups (Figure 2D).

## Discussion

Fontan associated liver disease is a form of hepatopathy progressing from mild liver test abnormalities in childhood (11, 12), to the liver fibrosis in a majority of older patients and liver cirrhosis in some patients (13, 14) or even rarely to hepatocellular carcinoma (15). Three main stages of FALD have been described as: (1) liver congestion, sinusoidal dilatation and perisinusoidal necrosis; (2) perisinusoidal fibrosis and liver nodules formation without portal hypertension; (3) advanced fibrosis and portal hypertension (6). FALD pathophysiology and structure is more similar to the Budd-Chiari syndrome than to other hepatopathies, as both are characterized by hepatic venous congestion and occurrence of liver nodules (16–18).

The ELF test, as a recognized direct biochemical liver fibrosis marker in various hepatopathies, showed poor correlation with MRI hemodynamic parameters in our study. Lack of correlation between ELF test and liver fibrosis was also observed in other FALD studies (19, 20). When we analyzed the ELF test components separately, in contrast to HA and tissue inhibitors of matrix metaloproteinases (TIMP-1), a significant correlation between PIIINP and some MRI parameters was found. Collagen III is the largest component of the extracellular matrix in the liver. During its synthesis, the N-terminal propeptide is cleaved from procollagen type III and released into blood as the direct marker of fibrogenesis (21). PIIINP is not specific just to the liver fibrosis, but it may be also increased in patients with myocardial hypertrophy or impaired diastolic ventricular dysfunction (22, 23), however, in our study no correlation between PIIINP and end-diastolic myocardial mass was observed.

A positive significant correlation between PIIINP and inferior vena cava flow (Qivc) in our study indicates that the progression of liver fibrosis increases inferior vena cava flow in Fontan patients. A similar observation between inferior vena cava flow assessed by MRI computational fluid dynamic and the collagen deposition from the liver biopsy specimens was recently published by Trusty et al. (24, 25).

Liver nodules are frequently observed in adults after the Fontan procedure with reported prevalence from 39 to 56% in a multicenter observational MRI/CT study (18). In our study were liver nodules diagnosed in 15/39 (38%) of patients. There was no difference between patients with and without liver nodules with regard to the basic perioperative risk factors such as age at TCPC, ventricular morphology, pulmonary artery pressure and resistance or size of the pulmonary arteries, except for longer cardiopulmonary bypass time in patients with liver nodules. There was also no difference at the time of study between both groups in age and interval from TCPC, body mass index, liver elastography, VO_2*max*_ at CPET, pulse oximetry at rest or exercise or quality of life (SF-36 questionnaire). The biochemical tests were also similar in both groups except for significantly elevated PIIINP in patients with nodules.

Our study showed that the occurrence of liver nodules had a significant impact on the hemodynamics assessed by MRI. The subgroup of patients with nodules had significantly increased systemic venous and aortic flow, end-diastolic volume and stroke volume and lower heart rate. Since in this subgroup there was a significant correlation between PIIINP and inferior vena cava flow, but not between heart rate and end-diastolic volume, we assume that in patients with liver nodules the end-diastolic volume was influenced mainly by an increased hepatic blood flow. On the contrary, there was no correlation between PIIINP and inferior vena cava flow in the subgroup of patients without the liver nodules. The overall contribution of aortopulmonary collateral flow and atrioventricular valve regurgitation fraction to the ventricular volume overload was not significant and similar in both subgroups.

The pathophysiology of an increased hepatic blood flow in Fontan patients can be explained initially by the hepatic arterial buffer response (HABR), a compensatory ability of the hepatic artery to increase flow in response to decreased portal flow (26), followed by subsequent gradual progression of the hepatic arterialization caused by the liver fibrosis and liver nodules formation (16, 18, 27). Indeed, in a detailed MRI study, a markedly increased hepatic arterial flow forming 45% of hepatic venous flow was observed in children after the Fontan procedure compared to non-significant arterial flow contribution to the hepatic venous flow in the control group (28). Interestingly, an increased cardiac output with elevated central venous pressure and preserved ventricular function was also associated with increased mortality in young adults after various types of Fontan procedure (29). The detrimental effects of liver fibrosis and cirrhosis caused by various hepatopathies on heart function are recognized and well described (30) but so far not specifically addressed in Fontan patients.

The clinical significance of liver nodules occurrence in our study, which are considered as markers of advanced stage of FALD, suggests that although these patients are asymptomatic, in a good clinical condition (NYHA I or II), with an acceptable quality of life, exercise capacity, arterial saturations and NTproBNP levels, they are at risk of inappropriate single ventricular volume due to an increased hepatic flow.

### Limitations

When planning the study we did not appreciate the importance of possible effects of liver fibrosis on Fontan circulation and therefore the inferior vena cava flow was measured only above the hepatic veins according to the usual protocols. Therefore we can present in our study only indirect evidence of negative influence of an increased hepatic flow on Fontan circulation caused by the liver fibrosis.

## Conclusion

Venous congestion, limited cardiac output and hypoxia trigger FALD initially after TCPC. Later on the gradual progression of the liver fibrosis, particularly hepatic arterialization caused by the liver nodules formation, increases inferior vena cava flow. Subsequent ventricular volume overload, which may further compromise already limited single ventricle functional reserve in Fontan adult patients, should be diagnosed and appropriately treated. PIIINP seems to be a good non-invasive marker of liver fibrosis in FALD, but further studies are needed.

## Data availability statement

The original contributions presented in this study are included in the article/Supplementary material, further inquiries can be directed to the corresponding author.

## Author contributions

VC and JJ contributed to conception and design of the study. VC wrote the manuscript and performed statistical analyses. DJ collected all clinical and laboratory data and organized spreadsheets for the statistical analyses. VT performed echocardiographic examinations and analyses. OM reviewed catheterization and angiocardiographic data. RG and RP reviewed surgical notes and perioperative clinical records. PA was responsible for the clinical examination and outpatient summary records. TA supervised magnetic resonance examinations and performed all MRI analyses. MŠ performed sonographic examination and elastographic analyses. VI supervised cardiopulmonary exercise tests and performed analyses. KK supervised and guarantied biochemical and ELF test laboratory examination. All authors contributed to manuscript revision, read, and approved the submitted version and there was no one else who fulfils the criteria that has been excluded as an author.
