# Supplementary material for: Impact of liver fibrosis and nodules formation on hemodynamics in young adults after total cavopulmonary connection. A magnetic resonance study
Source: Front Cardiovasc Med. 2022 Sep 29;9:986653. doi: 10.3389/fcvm.2022.986653 (PMC9558211; doi:10.3389/fcvm.2022.986653)
Supplement: Supplementary file 1 [file Data_Sheet_1.pdf]

Supplementary Table 1. Correlations between MRI variables and ELF test

MRI variables	Measure	PIIINP		TIMP-1		HA		ELF score	
		r	p value	r	p value	r	p value	r	p value
Heart rate (beats/min)	71 ± 13	-0.150	0.361	0.249	0.127	-0.133	0.421	-0.166	0.314
End-diastolic volume (mL/m^2)	92.3 (77.5 - 105.6)	0.328	0.042	-0.095	0.565	0.070	0.672	0.207	0.206
End-systolic volume (mL/m^2)	41.5 (34.1 - 54.5)	0.213	0.192	0.050	0.764	0.120	0.466	0.204	0.213
Stroke volume (mL/m^2)	48.5 ± 8.7	0.341	0.034	-0.108	0.511	-0.045	0.785	0.171	0.297
Cardiac index (L/min/m^2)	3.3 (3.0 – 3.7)	0.198	0.227	0.065	0.692	-0.183	0.265	-0.021	0.898
Ejection fraction	0.54 (0.48 – 0.59)	-0.008	0.964	-0.099	0.548	-0.005	0.975	-0.050	0.762
End-diastolic myocardial mass (g/m^2)	50.6 ± 15.9	0.216	0.198	-0.124	0.466	0.074	0.662	0.209	0.215
AO flow (L/min/m^2)	2.9 ± 0.4	0.196	0.233	0.146	0.374	-0.118	0.475	0.068	0.681
Systemic venous flow (L/min/m^2)	2.3 ± 0.4	0.445	0.005	0.282	0.083	-0.085	0.606	0.253	0.119
IVC flow (L/min/m^2)	1.6 ± 0.3	0.465	0.003	0.278	0.087	-0.073	0.658	0.248	0.127
SVC flow (L/min/m^2)	0.7 ± 0.1	0.219	0.180	0.176	0.284	-0.078	0.638	0.162	0.325
IVC / SVC flow	2.4 ± 0.5	0.192	0.241	0.065	0.694	0.017	0.919	0.062	0.710
Pulmonary artery flow (L/min/m^2)	2.2 (1.9 - 2.4)	0.179	0.277	0.202	0.217	0.069	0.674	0.120	0.466
Pulmonary venous flow (L/min/m^2)	2.9 ± 0.5	0.108	0.513	0.276	0.089	-0.077	0.640	0.056	0.735
Qcoll-pulm flow (L/min/m^2)	0.56 ± 0.24	-0.101	0.540	0.033	0.841	-0.040	0.808	-0.078	0.637
Qcoll-pulm/AO flow x 100 (%)	19 ± 7	-0.205	0.211	-0.014	0.933	0.026	0.874	-0.094	0.569
McGoon index	2.5 ± 0.7	-0.096	0.563	0.004	0.981	-0.005	0.976	-0.087	0.598
Nakata index (mm^2/m^2)	245 ± 56	-0.077	0.641	0.295	0.068	0.061	0.712	0.043	0.793

Data are represented as mean ± SD or median (IQR) and Pearson´s or Spearman´s coefficients as appropriate. AO, aorta; HA, hyaluronic acid; IVC, inferior vena cava; LPA, left pulmonary artery; PIIINP, procollagen III amino-terminal peptide; Qcoll-pulm, aortopulmonary collateral flow; RPA, right pulmonary artery; RPV, right pulmonary vein; SVC, superior vena cava; TIMP-1, inhibitors of matrixmetaloproteinases
